# Automated 3D Phenotype Analysis Using Data Mining

**DOI:** 10.1371/journal.pone.0001742

**Published:** 2008-03-05

**Authors:** Ilya Plyusnin, Alistair R. Evans, Aleksis Karme, Aristides Gionis, Jukka Jernvall

**Affiliations:** 1 Institute of Biotechnology, University of Helsinki, Helsinki, Finland; 2 Department of Geology, University of Helsinki, Helsinki, Finland; 3 Yahoo! Research Barcelona, Barcelona, Catalunya, Spain; University of Chicago, United States of America

## Abstract

The ability to analyze and classify three-dimensional (3D) biological morphology has lagged behind the analysis of other biological data types such as gene sequences. Here, we introduce the techniques of data mining to the study of 3D biological shapes to bring the analyses of phenomes closer to the efficiency of studying genomes. We compiled five training sets of highly variable morphologies of mammalian teeth from the MorphoBrowser database. Samples were labeled either by dietary class or by conventional dental types (e.g. carnassial, selenodont). We automatically extracted a multitude of topological attributes using Geographic Information Systems (GIS)-like procedures that were then used in several combinations of feature selection schemes and probabilistic classification models to build and optimize classifiers for predicting the labels of the training sets. In terms of classification accuracy, computational time and size of the feature sets used, non-repeated best-first search combined with 1-nearest neighbor classifier was the best approach. However, several other classification models combined with the same searching scheme proved practical. The current study represents a first step in the automatic analysis of 3D phenotypes, which will be increasingly valuable with the future increase in 3D morphology and phenomics databases.

## Introduction

Statistical analysis of shape is a fundamental problem that is frequently encountered in biology. In paleontology, the bulk of information about the prehistoric world is deduced from the shape of fossils, whereas in developmental genetics the phenotypic significance of genes and pathways is often inferred from shape abnormalities. Automatic detection of phenotypic features is hindered by the facts that 3D morphology is difficult to measure and that the theory behind the analysis of complex structures such as 3D surfaces or density maps is more or less in its infancy [1]. This makes shape an unfavorable source of information when compared to other variables that can be assessed through linear or sequential measurements (e.g. gene sequences). However, progress in 3D data acquisition technologies suggests that in the near future highly accurate and relatively inexpensive scanners will be widely available, which will inevitably lead to the accumulation of large amounts of 3D morphological data. To make effective use of these data there will be a need for fast automated methods for conducting searches and building descriptive and predictive models on selected data sets.

Unlike morphological data, other types of 3D data have been deposited in databases for some time, with examples ranging from 3D statue scans [2] to 3D CAD models [3] and biomolecular structures [4]. 3D data repositories have facilitated interest in analysis and retrieval by content of 3D shapes eventually leading to experimental implementations of several search engines [5]. However, these studies have not addressed the domain of biological 3D phenotypes, which presents a distinct case with its own problems and objectives. In this paper we propose a method for automated analysis and searching of 3D morphologies. In our method we combine data mining with the latest advances in dental shape research.

We selected mammalian teeth as the morphological system to focus on because these structures have several attractive properties: in any given species tooth shape is generally very consistent and tightly regulated by genes, tooth shape is adaptive and correlates with the physical properties of key dietary sources [6]–[8], tooth shape displays great variation across taxa [9]–[11], and dentitions are a classic example of the study of phenotypes with a long tradition of often experience-based visual classification schemes.

Statistical analysis of dental shape carried out until now can be coarsely grouped into three different approaches with the main difference being the type of model used to represent the shape of analyzed samples. In the most traditional approach, samples are compared by taking sets of linear measurements of defined local structures present on the surface of a tooth crown (e.g. shearing quotient [12], [13]). Another approach, termed geometric morphometric analysis, employs a shape model that consists of a finite set of surface points defined by local geometrical patterns [14]. These points are called landmarks and must be detected and measured from all objects that are compared. After detection, landmark sets are analyzed in a sequence of well-developed procedures. To eliminate non-shape variation, landmarks are superimposed by recursively minimizing a given scoring function such as the mean square distance between the corresponding points. After this, shape differences can be described directly by the distances between corresponding points or by partial warp scores measured by non-linearly transforming (warping) all landmark sets to a reference set. Both of these approaches place strict limitations on shape variability, since a given set of local structures or landmarks must be detected on all samples. Partly in response to these limitations, some researchers have recently adopted a set of tools, termed Geographic Information Systems (GIS), originally designed for landscape analysis. From a 2.5 dimensional surface model, termed a Digital Elevation Model (DEM), GIS can measure relief, surface orientation, drainage areas and other topological attributes. If one switches landscape data with dental shape data, the obtained parameters can be grouped into shape vectors which represent morphologies in subsequent analyses [15]–[20].

**Figure 1:**
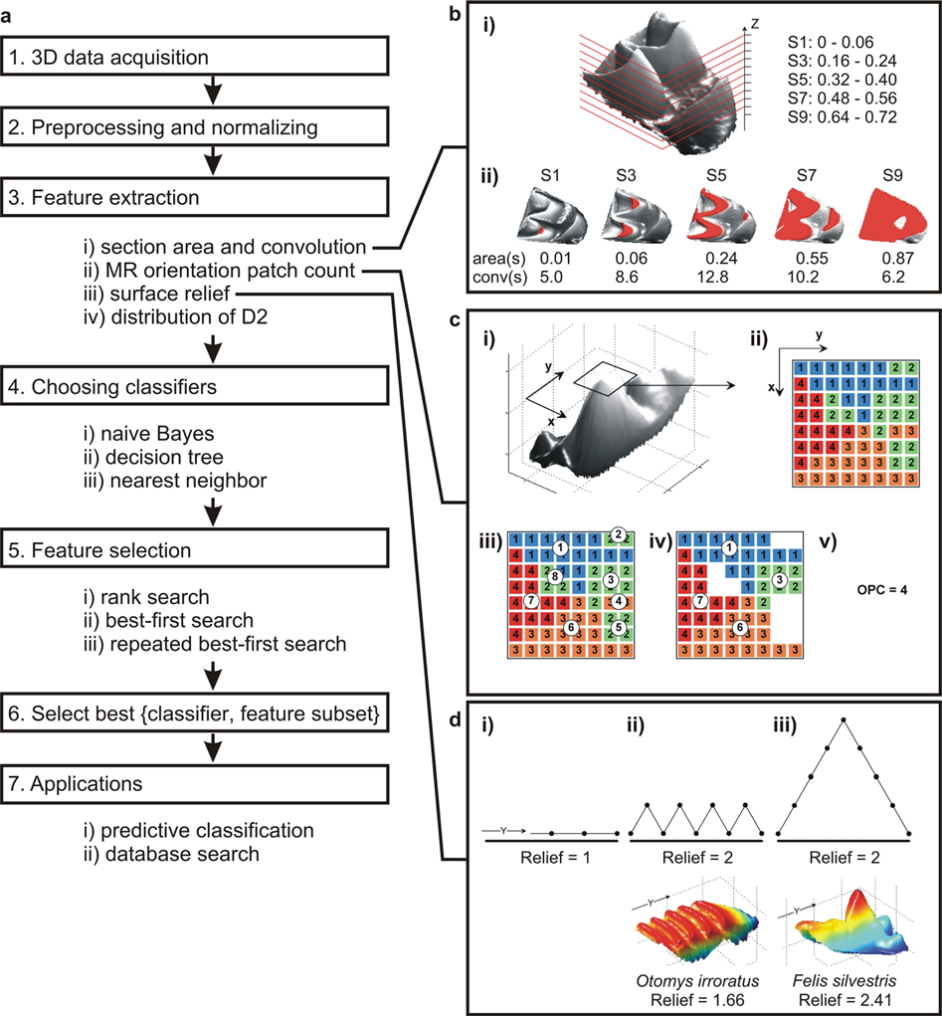
Outline of procedures for data mining of morphology. (a) Seven main steps from 3D data acquisition and processing, feature extraction, data mining procedures (classifiers and feature selection) to possible applications of the method. (b–d) illustrations of feature extraction methods. (b) Section areas and section convolutions. (i) Tooth (upper molar of *Rhinolophus blasii*) is divided into 10 equal sections perpendicular to the z-axis. Upper and lower bounds (relative to the z_Apex_) for every second section are given along the z-axis. (ii) Occlusal view with every second section highlighted in red, with areas and convolutions for each section. (c) Orientation patch count (OPC). (i) Surface of the tooth (upper molar of *Felis silvestris*) is grouped into surface vertices according to their orientation in the xy-plane (ii). (iii) Vertices are further grouped according to their 4-cell connectivity followed by (iv) exclusion of small patches. (v) The resulting OPC value is the final number of patches. (d) The effect of surface folding and elongation on surface relief. Relief is calculated by dividing the 3D surface area by its 2D projected area. A flat, unspecialized surface (i) has a relief of 1. If the surface is folded (ii), such as in *Otomys irroratus*, or elongated (iii), such as in *Felis silvestris*, its relief increases.

**Figure 2:**
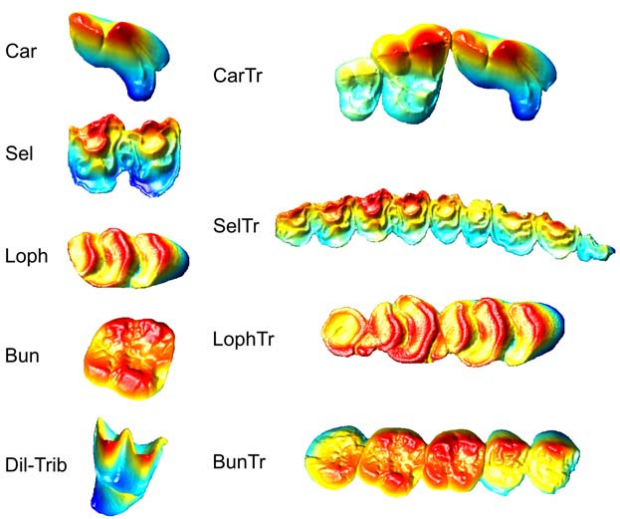
Dental types illustrated by samples from *tooth-morph* and *toothrow-morph* sets. Dental types: Car, carnassials; Dil-Trib, dilambdodont and tribosphenic; Sel, selenodont; Loph, lophodont; Bun, bunodont; CarTr, carnassial tooth row; SelTr, selenodont tooth row; LophTr, lophodont tooth row; BunTr, bunodont tooth row. Sample morphologies: Car, *Canis lupus* u-p4; Dil-Trib, *Rhinolophus blasii* u-m2; Sel, *Alcelaphus buselaphus* u-m2; Loph, *Berylmys bowersi* u-m1; Bun, *Pongo pygmaeus* u-m2; CarTr, *Canis lupus* u-p2m12; SelTr, *Alcelaphus buselaphus* u-tr; LophTr, *Berylmys bowersi* u-m1-3; BunTr, *Pongo pygmaeus* u-tr. u, upper; m, molar; p, premolar. Upper right teeth and tooth rows; anterior to the right.

Our objective was to design an automated method for building descriptive and/or predictive models given a set of 3D morphologies, and to use these models for searching 3D databases (Fig. 1). Since this immediately implied the use of a shape model that can be automatically constructed from any 3D object, we used the GIS approach for extracting topological attributes. Next, we applied feature selection algorithms from machine learning [21] to pick feature combinations that performed best at different tasks. We applied this approach to two different problems: the dietary prediction problem where the objective was to find combinations of dental features that worked best for distinguishing the teeth of animals in different dietary classes, and the morphotype prediction problem where the features were selected to discriminate between conventional dental types (carnassial, dilambdodont/tribosphenic, selenodont, lophodont and bunodont; Fig. 2). Both problems were evaluated on a data set of single teeth and a data set of tooth rows, and the dietary prediction problem was additionally evaluated on the mixed set of single teeth and tooth rows. Altogether this yielded five different cases.

We compiled five training sets of DEMs interpolated from 3D surface scans of upper cheek teeth of different mammalian species: *tooth-diet*, a set of 42 single teeth labeled by four dietary categories; *tooth-morph*, a set of 52 single teeth labeled by five dental types; *toothrow-diet*, a set of 36 tooth rows labeled by four dietary categories; *toothrow-morph*, a set of 36 tooth rows labeled by five dental types, and *mixed-diet*, the union of the *tooth-diet* and *toothrow-diet* sets. Compositions of the training sets are given in Table S1. Each DEM was mapped to a set of 100 topological features using MatLab procedures. We then coupled three different feature selection schemes (rank search, bidirectional best-first search and repeated bidirectional best-first search) with seven different classification models (three basic types: naive Bayes, decision tree and k-nearest neighbor) to perform a variety of data mining searches (Fig. 1). Under a 10-fold cross validation scheme, each search yielded a feature combination that is a local minimum for predicting dietary or dental type category under the classifier used for guiding the search. This combination of 21 data mining searches was performed on all training sets, with the exception of *mixed-diet* set for which we did not perform the repeated searches.

Using the classifiers obtained by the data mining, we constructed ‘ToothKit’, a stand-alone Java application for working with unannotated data sets of 3D morphologies. The application calculates the topological attributes for a new morphology and then classifies it according to one of the classifiers or finds other morphologies in a data set that are most ‘similar’ to the given morphology according to the selected classifier (see Discussion). This can be seen as a proof of concept to automatic phenotype analyzers for large 3D data sets.

## Results

Our results showed that for all five training sets the same basic set of features combined with an appropriate feature selection schemes yielded accurate classifiers in a very short time. In practice, this demonstrates that relationships between shape and categorical factors of interest can be extrapolated from a given training set to new data in an automated fashion. Search results (Table S2) were used to compare efficiency and accuracy of the examined searching options.

### Comparing feature selection schemes

Feature selection schemes were compared by averaging cross validation error rates (CVER), sizes of feature sets found and searching times across the classification models (Table S2). The search based on feature ranking had consistently lower performance than the best-first searches. When averaged across classifiers it was 8%, 7%, 11%, 6% and 6% less accurate than the best-first search, for the *tooth-diet*, *toothrow-diet*, *mixed-diet*, *tooth-morph* and *toothrow-morph* training sets respectively.

Repeating the best-first search from 50 random subsets improved performance only slightly: by 4%, 7%, 1% and 3% for the *tooth-diet*, *toothrow-diet*, *tooth-morph* and *toothrow-morph* training sets respectively. As expected, a best-first search initiated from an empty subset found on average the smallest subsets. Subsets found by repeatedly initiating a best-first search from random starting points were about two to three times larger and those found by rank search from two to four times larger.

The linear rank search was the fastest, with average searching times 14, 10, 34, 18 and 9 sec for the *tooth-diet*, *toothrow-diet*, *mixed-diet*, *tooth-morph* and *toothrow-morph* training sets respectively. Best-first search was at most twice as slow, with corresponding times being 23, 18, 72, 29 and 16 sec. Repeated best-first search was from 700 to 2500 times slower with average searching times ranging from two to six hours.

### Comparing classification models

1-NN (1-nearest neighbor) was consistently the best classifier for all five problems indicating the mosaic-like partition of the underlying feature space. Performances of other NN models varied across different problems from as good as 1-NN to several times less accurate. Differences between the best generative (naive Bays with multivariate kernel (NB-N) and normal (NB-K)) and the best non generative (C4.5 decision tree and NN) models were 5%, 8%, 12%, 5% and 4% for *tooth-diet*, *toothrow-diet*, *mixed-diet*, *tooth-morph* and *toothrow-morph* training sets respectively.

Accuracy (accuracy = 1–CVER) of the best classifiers was at least 85% for all five training sets. Best classifier for *toothrow-morph* training set was 97% accurate, for *tooth-morph* training set 94% accurate and for *tooth-diet*, *toothrow-diet* and *mixed-diet* training sets 93%, 91% and 85% accurate respectively. Performance on samples outside the training sets can be evaluated using the ToothKit software that is supplied with the best generative and non generative classifiers that are asterisked in Table S2 (see ToothKit).

### Comparing features

We were also interested in which features were used by different classification tasks. We assumed that this is best revealed by the probability distribution of features in the selected subsets averaged across all subsets selected for a given classification task. The probability distribution of a feature in a set is its frequency in that set (0 or 1) normalized by the length of the set. The top ten features ranked by their mean probability are given in Tables 1–5 and the number of matches between top ten features of different training sets are given in Table 6.

**Table 1:** Features used in the tooth-diet training set.

ID	Feature	P
99	D2dist-100000-mean	0.2
15	sectionConv −10 −5 −5	0.04
42	MROPC −5 −0.1 −9	0.04
22	MROPC −4 −0.002 −9	0.03
25	MROPC −4 −0.008 −9	0.03
57	MROPC −7 −0.006 −9	0.03
16	sectionConv −10 −5 −6	0.03
1	sectionAreas −10 −5 −1	0.03
73	MROPC −8 −0.06 −9	0.03
98	relief	0.02

Top ten features for the tooth-diet training set ranked by their mean probability distribution. ID, feature ID; Feature, procedure name including parameters; P, mean value for probability density function rounded to the nearest percent.

**Table 2 pone-0001742-t002:** Features used in the toothrow-diet training set.

ID	Feature	P
50	MROPC −6 −0.04 −9	0.1
6	sectionAreas −10 −5 −6	0.06
9	sectionAreas −10 −5 −9	0.06
4	sectionAreas −10 −5 −4	0.05
93	MROPC −10 −0.02 −9	0.04
5	sectionAreas −10 −5 −5	0.04
61	MROPC −7 −0.04 −9	0.04
3	sectionAreas −10 −5 −3	0.04
13	sectionConv −10 −5 −3	0.03
7	sectionAreas −10 −5 −7	0.03

Top ten features for the toothrow-diet training set ranked by their mean probability distribution. See Table 1 for definitions.

**Table 3 pone-0001742-t003:** Features used in the mixed-diet training set.

ID	Feature	P
99	D2dist-100000-mean	0.11
7	sectionAreas −10 −5 −7	0.1
2	sectionAreas −10 −5 −2	0.07
42	MROPC −5 −0.1 −9	0.04
1	sectionAreas −10 −5 −1	0.03
6	sectionAreas −10 −5 −6	0.03
9	sectionAreas −10 −5 −9	0.03
54	MROPC −7 −0.001 −9	0.03
11	sectionConv −10 −5 −1	0.03
15	sectionConv −10 −5 −5	0.03

Top ten features for the mixed-diet training set ranked by their mean probability distribution. See Table 1 for definitions.

**Table 4 pone-0001742-t004:** Features used in the tooth-morph training set.

ID	Feature	P
24	MROPC −4 −0.006 −9	0.07
6	sectionAreas −10 −5 −6	0.07
11	sectionConv −10 −5 −1	0.06
2	sectionAreas −10 −5 −2	0.06
100	D2dist-100000-std	0.05
25	MROPC −4 −0.008 −9	0.05
4	sectionAreas −10 −5 −4	0.05
7	sectionAreas −10 −5 −7	0.04
99	D2dist-100000-mean	0.04
73	MROPC −8 −0.06 −9	0.04

Top ten features for the tooth-morph training set ranked by their mean probability distribution. See Table 1 for definitions.

**Table 5:** Features used in the toothrow-morph training set.

ID	Feature	P
15	sectionConv −10 −5 −5	0.06
3	sectionAreas −10 −5 −3	0.06
54	MROPC −7 −0.001 −9	0.05
65	MROPC −8 −0.001 −9	0.05
4	sectionAreas −10 −5 −4	0.05
95	MROPC −10 −0.06 −9	0.05
39	MROPC −5 −0.04 −9	0.05
62	MROPC −7 −0.06 −9	0.04
43	MROPC −6 −0.001 −9	0.04
80	MROPC −9 −0.008 −9	0.03

Top ten features for the toothrow-morph training set ranked by their mean probability distribution. See Table 1 for definitions.

**Table 6:** Overlap of features in the five training sets.

	tooth-diet				
		toothrow-diet			
			mixed-diet		
				tooth-morph	
					toothrow-morph
**tooth-diet**	x	0	4	3	1
**toothrow-diet**		x	3	3	2
**mixed-diet**			x	5	2
**tooth-morph**				x	1
**toothrow-morph**					x

The number of exact matches between top ten features of different training sets.

The top ten feature sets were distinct for each of the five classification problems with only partial overlaps. There were no common features between *tooth-diet* and *toothrow-diet* top tens, but, quite expectedly, 4 and 3 common features between top tens of these sets and the mixed set respectively. This appears to be good evidence of the repeatability of feature selection. Top ten features for dietary prediction and dental type prediction had several matches. This is particularly the case for the *mixed-diet* problem (Table 3) where half of the top ten features matched those of the *tooth-morph* problem (Table 4). Of the 50 features in the top ten sets 31 were unique, indicating a wide exploitation of the underlying feature pool.

## Discussion

Here, we have demonstrated how the analysis of 3D phenotypes can be automated: the researcher selects phenotypes for the training set, converts them to DEMs, labels samples by the categorical variable of interest, extracts features, runs feature selection and applies the obtained classifiers to new samples. Notably, most of these steps are automated, where only the selection and labeling of the training set need to be carried out by the investigator. The selection of samples can be further facilitated by gathering 3D phenotypes into databases, which has already started to happen [22]–[24] (also see http://www.digimorph.org/).

A couple of the features show distinctly higher occurrences in the training sets. D2dist mean (mean distance between two randomly selected points; see Methods) is by far the most frequent for the tooth-diet problem (and most frequent for the mixed-diet problem), which appears to indicate some differentiation in this feature with diet. For the toothrow-diet, a measure of MROPC (orientation patch count; see Methods) is the highest, which follows earlier findings of a correlation between OPC and diet [19]. The most frequent features for the other problems are a mixture of MROPC, section areas and section convolutions. The surface relief feature has the lowest frequency of the four basic types of feature extraction. Overall, it seems that no single feature is outstandingly useful in the classification problems, and it is the combination of features that is the most important in categorizing the morphologies.

Comparisons of feature selection schemes, classification models and features revealed several important aspects. Compared to feature ranking, direct evaluation of feature subsets produced clearly better classifiers at a moderate cost of approximately doubling the searching time. Repeating the best-first search, on the other hand, does not appear beneficial as a thousand-fold increase in computation time is traded for only a moderate improvement in accuracy. NN models were consistently the best for all five problems, and were on average two times better than the generative NB models. However, in terms of absolute accuracies both NB and Tree/NN models performed well for all but the mixed set, with CVER ranging from 7% to 15% and from 3% to 9% respectively. Presumably due to its large size, *mixed-diet* was a difficult set for both NB and tree/NN models, with the best classifiers scoring 27% and 15% respectively. A comparison of features used by different classification tasks showed that the designed feature pool was exploited to large extent (31 of the 50 top ten features were unique) and that, in spite of partial overlaps, different problems favored different features.

There are at least two straight-forward applications for the classifiers we obtained. First, the classification model can be used to extrapolate relationships between shape and the variable of interest from the training set to new instances. This has immediate applications in paleontology where classifiers built on extant species can be used to reconstruct diet and other variables for extinct species. Moreover, such reconstructions will be objective as they will not include subjective assessments beyond the selection of the training sets. Another application is developmental biology where one central aim is to link abnormal shapes to underlying developmental mechanisms, and our approach can be used together with forward genetics to screen new phenotypes for which the underlying development is not yet known.

Second, using these classifiers we can implement retrieval by content for unannotated databases of 3D phenotypes, i.e. finding the *k* morphologies in the database that are most similar to either a specific query or specific query morphology [21]. Examples of such queries include: searching for extant dental morphologies that are most likely to be specialized in the same diet as a given extinct sample; finding mutant phenotypes that are most likely to be linked to a particular mutation; or simply finding the morphologies that are most similar in shape to a given query morphology. These types of queries are based on the measure of similarity, and classification can be used to define that measure [21]. We can return all objects that are predicted to be in the same category as the query morphology, or we can extend this approach by defining similarity as a function of posterior probabilities. For example
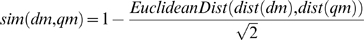
(1)where *dm* is the database morphology, *qm* the query morphology, and *dist* the posterior distribution assigned by the employed classifier. To illustrate this idea, ToothKit implements a search function that allows the user to search a disk using any of the classifiers supplied with the program and the similarity measure as defined in the above equation. Preliminary tests using *tooth-morph* and *toothrow-morph* classifiers suggest that these searching schemes return morphologies that are considered relevant in terms of general shape similarity. In future studies, performance can be assessed more formally by precision-recall and other measurements.

Whereas our study brings automated approaches into the domain of biological shape analysis, it obviously does not provide a foolproof technique for all morphologies or classification tasks. Biological 3D objects, which vary greatly among species and from individual to individual, are especially challenging for statistical comparison and major advances in theory of this field are needed before any two 3D surfaces or solids can be compared within a universal framework. However, this study does demonstrate that for a limited domain of 3D shapes it is possible, and actually quite straightforward, to define a set of features that will provide a basis for automated statistical analysis. In addition to teeth, GIS-like feature extraction is applicable to many other objects that approximate 2.5D surfaces, both biological [turtle shells, beetle carapaces, mollusk shells, faces, bird beaks, insect eyes, skin wrinkling, joint surfaces, fish body shapes and footprints] and non-biological (land forms). Whereas many of the features used on teeth to build classifiers may be applicable to other shapes, it remains to be seen how successful these features would be at classifying diverse biological shapes. Interestingly the D2 shape function (the mean distance between two random surface points), which was successfully incorporated into several classifiers, was adapted from a general purpose comparison approach designed to discriminate between 3D objects that have nothing to do with teeth (such as CAD models for cars). This suggests that features do not have to be system-specific. On the other hand, the frequently used features in classifications may be helpful in identifying functionally significant aspect of morphology. For example, the OPC was previously suspected to be informative in distinguishing diet [19], which is now supported by our analysis.

Yet another consideration is the efficiency of automated 3D analysis in comparison to trained persons. Our informal tests suggest that expert opinion (with over 20 years' experience) is at least as accurate as our automated routines. However, expert opinions do not provide implicit probabilities and are limited to a small set of (traditional) classification problems. Additionally, with an increasing number of teeth, the automated routines becomes more efficient, especially if we consider that each new classification problem would require manual reinspection of each specimen by a person. Therefore, we envision that these new types of morphological analyses promise not only informative ways for classifying morphologies, but may also be useful in exploring classic questions of morphospace occupation, and tempo and mode in morphological evolution (e.g. [9], [31], [32]).

When implemented in conjunction with a 3D morphological database, this approach has the potential to boost the application of 3D shape analysis, since even non-experts will gain the ability to build “on the fly” descriptive/predictive models from arbitrary sets of 3D scans. Advances along these lines will push the development of 3D morphology analysis towards the ease of the current sequence analysis.

## Methods

### Classifying samples

For *tooth-diet* and *toothrow-diet* sets, morphologies were labeled according to the dietary preferences of the corresponding species (estimated from the literature [25]): vertebrates (V), invertebrates (I), grass/foliage (G–F), and seeds/fruits/succulent plant tissue (S-F-SPT). In *tooth-morph* samples were labeled by conventional dental types [10], [11]: carnassials (Car), dilambdodont and tribosphenic (Dil-Trib), selenodont (Sel), lophodont (Loph), and bunodont (Bun). For *toothrow-morph*, samples were labeled by the dominant dental type: carnassial tooth row (CarTr), selenodont tooth row (SelTr), lophodont tooth row (LophTr), and bunodont tooth row (BunTr). The test sets were comprised of teeth from members of the Orders Artiodactyla, Carnivora, Chiroptera, Perissodactyla, Primates and Rodentia (see Table S1 for full species list).

### Digitizing morphologies

In the following text we use a Cartesian coordinate system, with x standing for buccal to lingual, y for posterior to anterior and z for cervical to occlusal vectors. A_proj_ refers to the xy-projection area of the morphology.

Samples were scanned using Nextec Hawk 3D laser scanner and Roland Dr Picza needle scanner at between 10 and 50 µm resolution, depending on the size of the sample. Scans were added for interactive viewing in our online database MorphoBrowser (see http://morphobrowser.biocenter.helsinki.fi/). Before scanning, teeth were oriented manually to maximize crown-base projection in the xy-plane. Scanning produced unordered point cloud files that were further processed to digital elevation models (DEMs). A 2D outline of the single tooth/tooth row was selected to separate the analyzed surface from the background of the maxilla bone and the fixative material. This was done by picking a sequence of boundary points using Surfer Version 8 software. Boundary points were then used to delete the background part of the point cloud in Matlab Version 7. The remaining surface points were interpolated to DEM using triangle-based cubic interpolation implemented in Matlab *griddata* function. Artifacts in concave regions of the surface boundary produced during surface interpolations based on convex hulls were deleted using boundary points. The total resolution of DEMs (number of rows×number of columns) was set to be approximately 10 000 points for single teeth and 50 000 points for tooth rows by setting digital grid resolution as a function of the dimensions of the point cloud's bounding box:
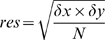
(2)where *delta x* and *delta y* are the point cloud's bounding box dimensions in the xy-plane, *N* is 10 000 or 50 000 depending on the sample, and *res* is the resolution of the digital grid.

Pilot studies using various resolutions were undertaken to find a middle resolution that was not so low as few of the major features were not present (such as cusps and crests), but not so high as to either give greater emphasis to very small features or to slow down scan processing. For other data types (e.g. skin wrinkling or joint surface shape), other resolutions may be more applicable.

### Feature extraction

To extract features we implemented five Matlab procedures with each procedure designated to a particular feature type. The exact definition of a feature could be modified by varying values of input parameters. Using these, we extracted 100 features (for the full list of feature names and IDs see Table S3). Procedures were designed to yield features invariant to translation, scale, reflection, and rotation in the xy-plane.

The initial set for this study was chosen after experimentation with a larger number of different features. In these pilot studies, several of the features were not found to be informative for the classification tasks and so were excluded from the current study, with the most informative ones being retained. For different types of data or classification tasks, these rejected features could be tested; however, we believe that much of the variation in 2.5D shapes will be captured by the features used here.

### Section area and convolution (features 1–10 and 11–20 respectively)

Section areas were calculated by slicing the tooth in the xy-plane along the z axis. Starting from the maximum z value z_Apex_ and ending at the point
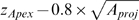
(3)morphologies were divided into 10 main sections, and the area of each main section was calculated as the mean of 5 subsections (Fig. 1*B*). Returned values were normalized by the xy-projection area.

For each section we also calculated convolution as defined by
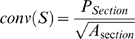
(4)where P_Section_ is the summed perimeter, and A_Section _the summed area, of all 2D objects in a given section. Convolutions of the main sections were averaged in the same fashion as the area.

These features are referred using “*sectionAreas* -*p1* -*p2* -*p3*” or “*sectionConv -p1 -p2 -p3*” syntax, where the name of the procedure is followed by the number of main sections (*p1*), the number of subsections (*p2*) and the index of the section examined (*p3*).

### Maximum of Rotated Orientation Patch Count (MROPC, features 21–97)

OPC is a measure of surface discontinuity that is quantified as the number of surface patches with different orientations in the xy-plane (Fig. 1*C*). This measure has been shown to correlate with the amount of mechanical processing of food required in both carnivorans and rodents [19]. OPC was calculated in four steps. First, surface normals were calculated for all surface vertices. Second, surface vertices were divided into a given number of orientation classes (*noc*) based on the angle between the xy-component of the corresponding normal and the reference vector. Third, vertices belonging to the same orientation class were clustered into patches according to 4-cell connectivity. Finally, patches smaller then a threshold value of minps*A_proj_ were discarded, where minps is the minimum patch size, and the number of the remaining patches was returned as the OPC.

To improve invariance against rotation in the xy-plane several OPC values were calculated by iteratively shifting the reference vector counterclockwise. The reference vector, which initially points along the x-axis, was iteratively shifted by 9 degrees (the *da* parameter) counterclockwise until it was shifted to the next orientation class as defined by the reference vector's initial direction. Examinations of the variance for *mixed-diet* showed that the maximum value from this rotation series is less affected by rotation (data not shown), hence we used the Maximum of Rotated Orientation Patch Count (MROPC).

We covered a wide range of MROPC definitions by iterating *noc* from 4 to 10 and *minps* through values 0.001, 0.002, 0.004, 0.006, 0.008, 0.01, 0.02, 0.04, 0.06, 0.08 and 0.1, producing 7×11 = 77 variations. These features are referred using “*MROPC-noc-mips-da*” syntax.

In this study, the *da* variable is constant and is merely shown to make feature definition transparent. The parameters *minps* and *noc* have a crucial effect on the type of patches that are counted. For a surface with large spatial variance, patch count will grow as a function of *noc* since *noc* defines the angular resolution at which neighboring patches can be discriminated. The rate of change for the patch count relative to *noc* is thus dependent on the spatial variance of the surface at different scales. This can explain why several MRPOC measures are selected: they capture the rate of change or the boundary resolution beyond which no change occurs. *minps* values, on the other hand, affect the type of patches that the patch count represents: for small values, any patches are counted, whereas for larger values, only large and probably flat ones are. However, we have not investigated in detail the behavior of MROPC as a function of its parameters.

Note that even though MROPC are more numerous than other descriptors, this does not produce bias towards selecting this feature type. Best first search feature selection algorithm examines all features during each selection step, and thus is not biased by the share number of any particular type. On the other hand, numerous combinations are more likely to produce informative features than less numerous, so one can expect that the selected MROPC are better “tuned” than the other types.

### Surface relief (feature 98)

Surface relief is defined as the 3D surface area divided by the xy-projection area (Fig. 1*D*). This measure is assumed to have functional significance, since it describes the amount of deviation from an unspecialized flat surface. Relief has been shown to vary with dietary adaptation in hominid apes [20]. This feature is referred as *relief*.

### D2dist (features 99 and 100)

These features comprise the mean and standard deviation of the distribution of D2 shape function. D2 measures the distance between two surface points chosen randomly with respect to the xy-plane. This measure was adapted from the work of Osada and coworkers, who proposed this method for comparing arbitrary 3D polygonal models [26]. They found that the dissimilarities between distributions of shape functions (particularly D2) provide a robust measure for discriminating between classes of objects (e.g., cars versus airplanes) in a moderately-sized database, despite the presence of arbitrary translations, rotations, scales, mirrors and model degeneracies.

In order to apply this method to dental DEMs we made several adjustments. First, points were sampled randomly with respect to the xy-plane instead of the object surface. Second, instead of representing D2 distributions with a piecewise linear function, distributions were represented only by their mean and standard deviation values. Third, linear distances were normalized by the square root of *A_proj_*.

These features are referred using “*D2dist-n-s*” syntax, where *n* is the size of the sample taken to estimate the distribution and *s* is the type of statistic calculated (mean or s.d.).

### Data mining tools

To perform data mining we used algorithms for probabilistic classification, model validation and feature selection, implemented in an open source WEKA library [27]. All data mining was performed using WEKA classes running on Java Platform (Standard Edition, Version 6), Microsoft Windows XP, on a PC with a 1.70 GHz CPU.

### Classification models

We used three types of probabilistic classification models: naive Bayes [28], decision tree and k-nearest neighbor [29]. Naive Bayes classifiers were built using *weka.classifiers.bayes.NaiveBayes*, with both normal and kernel density functions. Decision trees were built using C4.5 algorithm [30] implemented in *weka.classifiers.trees.J48.* To construct k-nearest neighbor models we used *weka.classifiers.lazy.Ibk.* Number of neighbors was set to 1, 3, 5 and 7, with neighborhood defined by Euclidean distance.

### Validating classifiers

Cross validation is a measure of model accuracy and its robustness against random changes in the relative composition of the training data and the data for which an inference is made [21]. In this work we coupled 10-fold cross validation, implemented in *weka.attributeSelection.WrapperSubsetEval*, with the seven classification models described above.

### Feature selection

In machine learning it is well recognized that the exact feature combination used for a particular classification task is generally the single most influential factor affecting classification accuracy [21]. In this work we compared the performance of three feature selection schemes: (1) rank search, (2) bidirectional best-first search and (3) repeated bidirectional best-first search. The first scheme ranks attributes according to their information gain, and then examines the cross validation error rate of incrementally increasing sets of attributes starting from the most informative (i.e. the best attribute, the best attribute and the second best, etc.). The second scheme was a conventional bidirectional best-first search initiated from an empty subset. The third option was a bidirectional best-first search repeatedly initiated from 50 subsets of random size and composition.

For the evaluation of feature subsets all searches used 10-fold cross validation combined with one of the seven classifiers. We used rank search *weka.attributeSelection.RankSearch* coupled to information gain measure *weak.attribureSelection.InfoGainAttributeEval* and the best-first search *weka.attributeSelection.BestFirst*.

### ToothKit

In this freeware Java program we implemented some of the major functionalities discussed above: converting *.stl, *.obj, *.dxf and *.wrl polygon meshes into DEMs, feature extraction, classification and retrieval by content. ToothKit is supplied with trained classifiers asterisked in Table S2. ToothKit is available from http://www.biocenter.helsinki.fi/bi/evodevo/toothkit/index.html.

## Supporting Information

Table S1 Composition of the training sets.
(0.24 MB DOC)

Table S2 Comparing feature selection schemes and classification models.
(0.11 MB DOC)

Table S3 Feature names and IDs.
(0.09 MB DOC)
