# Supplementary material for: Automated 3D Phenotype Analysis Using Data Mining
Source: PLoS One. 2008 Mar 5;3(3):e1742. doi: 10.1371/journal.pone.0001742 (PMC2254194; doi:10.1371/journal.pone.0001742)
Supplement: Table S1 — Composition of the training sets. (0.24 MB DOC) [file pone.0001742.s001.doc]

**Supplementary Information – Table S1**

Supplementary information for Plyusnin et al. (2008), Automated 3D phenotype analysis using data mining.

**Table S1.** Composition of the training sets.

Orders: Carn, Carnivora; Chir, Chiroptera; Rod, Rodentia; Artio, Artiodactyla; Peris, Perissodactyla; Prim, Primates. Diets: V, vertebrates; I, invertebrates; G-F, grass/foliage; S-F-SPT, seeds/fruits/succulent plant tissue. Dental types: Car, carnassials; Dil-Trib, dilamblodont and tribosphenic; Sel, selenodont; Loph, lophodont; Bun, bunodont; CarTr, carnassial tooth row; SelTr, selenodont tooth row; LophTr, lophodont tooth row; BunTr, bunodont tooth row. Teeth: u, upper; tr, cheek tooth row; p, premolar; m, molar.

| **tooth-diet** | | | |  | **toothrow-diet** | | | |
| --- | --- | --- | --- | --- | --- | --- | --- | --- |
| **class** | **order** | **species** | **teeth** |  | **class** | **order** | **species** | **teeth** |
| V | Carn | *C. aureus* | u-p4 |  | V | Carn | *C. aureus* | u-p4m12 |
|  |  | *C. lupus* | u-p4 |  |  |  | *C. lupus* | u-p4m12 |
|  |  | *F. silvestris* | u-p4 |  |  |  | *F. silvestris* | u-p4m1 |
|  |  | *G. genetta* | u-p4 |  |  |  | *G. genetta* | u-p4m12 |
|  |  | *G. gulo* | u-p4 |  |  |  | *G. gulo* | u-p4m1 |
|  |  | *L. lynx* | u-p4 |  |  |  | *L. lynx* | u-p4m1 |
|  |  | *M. lutreola* | u-p4 |  |  |  | *M. lutreola* | u-p4m1 |
|  |  | *M. martes* | u-p4 |  |  |  | *M. martes* | u-p4m1 |
|  |  | *V. vulpes* | u-p4 |  |  |  | *V. vulpes* | u-p4m12 |
|  |  | *V. zibetha* | u-p4 |  |  |  | *V. zibetha* | u-p4m12 |
| I | Chir | *C. gouldii* | u-m2 |  | I | Rod | *G. valdivianus* | u-m1-3 |
|  |  | *E. fuscus* | u-m2 |  |  |  | *I. stolzmanni* | u-m1-3 |
|  |  | *H. diadema* | u-m2 |  |  |  | *Oxymycterus sp.* | u-m1-3 |
|  |  | *M. ater* | u-m2 |  | G-F | Artio | *A. alces* | u-tr |
|  |  | *N. macrotis* | u-m2 |  |  |  | *A. buselaphus* | u-tr |
|  |  | *P. townsendii* | u-m2 |  |  |  | *H. meinertzhageni* | u-tr |
|  |  | *R. blasii* | u-m2 |  |  | Carn | *A. fulgens* | u-p4m12 |
|  | Rod | *G. valdivianus* | u-m1 |  |  | Peris | *E. burchelli* | u-tr |
|  |  | *I. stolzmanni* | u-m1 |  |  |  | *D. sumatrensis* | u-m1-3 |
|  |  | *Oxymycterus sp.* | u-m1 |  |  | Prim | *G. gorilla* | u-tr |
| G-F | Artio | *A. alces* | u-m2 |  |  | Rod | *H. goliath* | u-m1-3 |
|  |  | *A. buselaphus* | u-m2 |  |  |  | *M. rothschildi* | u-m1-3 |
|  |  | *H. meinertzhageni* | u-m2 |  |  |  | *O. irroratus* | u-m1-3 |
|  | Carn | *A. fulgens* | u-m2 |  | S-F- SPT | Artio | *P. porcus* | u-tr |
|  | Peris | *D. sumatrensis* | u-m2 |  |  |  | *S. scrofa* | u-tr |
|  |  | *E. burchelli* | u-m2 |  |  |  | *T. pecari* | u-tr |
|  | Prim | *G. gorilla* | u-m2 |  |  | Carn | *P. hermaphroditus* | u-p4m12 |
|  | Rod | *H. goliath* | u-m1 |  |  |  | *P. lotor* | u-p4m12 |
|  |  | *M. rothschildi* | u-m1 |  |  |  | *U. arctos* | u-p4m12 |
|  |  | *O. irroratus* | u-m1 |  |  | Prim | *P. pygmaeus* | u-tr |
| S-F- SPT | Artio | *P. porcus* | u-m2 |  |  |  | *P. troglodytes* | u-tr |
|  |  | *S. scrofa* | u-m2 |  |  |  | *P. ursinus* | u-tr |
|  |  | *T. pecari* | u-m2 |  |  | Rod | *A. hindei* | u-m1-3 |
|  | Carn | *P. hermaphroditus* | u-m1 |  |  |  | *N. mitchelli* | u-m1-3 |
|  |  | *P. lotor* | u-m1 |  |  |  | *P. maniculatus* | u-m1-3 |
|  |  | *U. arctos* | u-m2 |  |  |  |  |  |
|  | Prim | *P. pygmaeus* | u-m2 |  |  |  |  |  |
|  |  | *P. troglodytes* | u-m2 |  |  |  |  |  |
|  |  | *P. ursinus* | u-m2 |  |  |  |  |  |
|  | Rod | *A. hindei* | u-m1 |  |  |  |  |  |
|  |  | *N. mitchelli* | u-m1 |  |  |  |  |  |
|  |  | *P. maniculatus* | u-m1 |  |  |  |  |  |

| **tooth-morph** | | | |  | **toothrow-morph** | | | |
| --- | --- | --- | --- | --- | --- | --- | --- | --- |
| **class** | **order** | **species** | **teeth** |  | **class** | **order** | **species** | **teeth** |
| Car | Carn | *C. aureus* | u-p4 |  | CarTr | Carn | *A. lagopus* | u-p4m12 |
|  |  | *C. lupus* | u-p4 |  |  |  | *C. aureus* | u-p4m12 |
|  |  | *F. silvestris* | u-p4 |  |  |  | *C. lupus* | u-p4m12 |
|  |  | *G. genetta* | u-p4 |  |  |  | *F. silvestris* | u-p4m1 |
|  |  | *G. gulo* | u-p4 |  |  |  | *G. genetta* | u-p4m12 |
|  |  | *L. lynx* | u-p4 |  |  |  | *G. gulo* | u-p4m1 |
|  |  | *M. lutreola* | u-p4 |  |  |  | *L. lynx* | u-p4m1 |
|  |  | *M. martes* | u-p4 |  |  |  | *M. lutreola* | u-p4m1 |
|  |  | *V. vulpes* | u-p4 |  |  |  | *M. martes* | u-p4m1 |
|  |  | *V. zibetha* | u-p4 |  |  |  | *V. vulpes* | u-p4m12 |
| Dil-Trib | Carn | *P. townsendii* | u-m2 |  | SelTr | Artio | *A. alces* | u-tr |
|  |  | *R. blasii* | u-m2 |  |  |  | *A. buselaphus* | u-tr |
|  |  | *V. vulpes* | u-m1 |  |  |  | *C. elaphus* | u-tr |
|  | Chir | *A. lagopus* | u-m1 |  |  |  | *C. hircus* | u-tr |
|  |  | *C. gouldii* | u-m2 |  |  |  | *L. glama* | u-tr |
|  |  | *E. fuscus* | u-m2 |  |  |  | *O. aries* | u-tr |
|  |  | *H. diadema* | u-m2 |  |  |  | *R. tarandus* | u-tr |
|  |  | *M. ater* | u-m2 |  |  |  | *S. caffer* | u-m1-3 |
|  |  | *N. macrotis* | u-m2 |  | LophTr | Peris | *D. sumatrensis* | u-m1-3 |
|  |  | *O. megalotis* | u-m1 |  |  |  | *E. burchelli* | u-tr |
| Sel | Artio | *A. alces* | u-m2 |  |  |  | *T. terrestris* | u-m1-3 |
|  |  | *A. buselaphus* | u-m2 |  |  | Rod | *A. imitator* | u-m1-3 |
|  |  | *B. bonasus* | u-m2 |  |  |  | *B. bowersi* | u-m1-3 |
|  |  | *C. elaphus* | u-m2 |  |  |  | *H. brasiliensis* | u-m1-3 |
|  |  | *C. hircus* | u-m2 |  |  |  | *M. natalensis* | u-m1-3 |
|  |  | *G. camelopardalis* | u-m2 |  |  |  | *O. hypoxanthus* | u-m1-3 |
|  |  | *L. glama* | u-m2 |  | BunTr | Artio | *B. babyrussa* | u-tr |
|  |  | *O. aries* | u-m2 |  |  |  | *P. porcus* | u-tr |
|  |  | *R. tarandus* | u-m2 |  |  |  | *S. scrofa* | u-tr |
|  |  | *S. caffer* | u-m2 |  |  | Carn | *A. fulgens* | u-p4m12 |
| Loph | Peris | *D. sumatrensis* | u-m2 |  |  |  | *P. hermaphroditus* | u-p4m12 |
|  |  | *E. burchelli* | u-m2 |  |  |  | *U. americanus* | u-p4m12 |
|  |  | *E. zebra* | u-m2 |  |  |  | *U. arctos* | u-p4m12 |
|  |  | *T. indicus* | u-m2 |  |  | Prim | *G. gorilla* | u-tr |
|  |  | *T. terrestris* | u-m2 |  |  |  | *P. pygmaeus* | u-tr |
|  | Rod | *A. imitator* | u-m1 |  |  |  | *P. troglodytes* | u-tr |
|  |  | *B. bowersi* | u-m1 |  |  |  |  |  |
|  |  | *H. brasiliensis* | u-m1 |  |  |  |  |  |
|  |  | *M. natalensis* | u-m1 |  |  |  |  |  |
|  |  | *O. hypoxanthus* | u-m1 |  |  |  |  |  |
| Bun | Artio | *B. babyrussa* | u-m2 |  |  |  |  |  |
|  |  | *P. porcus* | u-m2 |  |  |  |  |  |
|  |  | *T. pecari* | u-m2 |  |  |  |  |  |
|  |  | *S. scrofa* | u-m2 |  |  |  |  |  |
|  | Carn | *A. fulgens* | u-m2 |  |  |  |  |  |
|  |  | *P. hermaphroditus* | u-m1 |  |  |  |  |  |
|  |  | *U. americanus* | u-m2 |  |  |  |  |  |
|  |  | *U. arctos* | u-m2 |  |  |  |  |  |
|  | Prim | *G. gorilla* | u-m2 |  |  |  |  |  |
|  |  | *P. pygmaeus* | u-m2 |  |  |  |  |  |
|  |  | *P. troglodytes* | u-m2 |  |  |  |  |  |
|  |  | *P. ursinus* | u-m2 |  |  |  |  |  |
