# Supplementary material for: Automated 3D Phenotype Analysis Using Data Mining
Source: PLoS One. 2008 Mar 5;3(3):e1742. doi: 10.1371/journal.pone.0001742 (PMC2254194; doi:10.1371/journal.pone.0001742)
Supplement: Table S2 — Comparing feature selection schemes and classification models. (0.11 MB DOC) [file pone.0001742.s002.doc]

**Supplementary Information – Table S2**

Supplementary information for Plyusnin et al. (2008), Automated 3D phenotype analysis using data mining.

**Table S2.** Comparing feature selection schemes and classification models.

Cross validation error rates, the size of the feature sets found and the time taken to run the searches for all combinations of training sets, feature selection schemes and classification models. Mean values across the classification models are given in the right-most column. Bold values are for best classifiers and asterisked values for classifiers included in ToothKit. Classifiers: NB-N, naive Bayes with multivariate normal; NB-K, naive Bayes with multivariate kernel; C4.5Tree, decision tree; k-NN, k-nearest neighbor. CVER, cross validation error rate rounded to the nearest percent; size, number of features in model; time, min:sec or hr:min:sec to run the search.

|  |  |  | **Classifier** | | | | | | | |  | |
| --- | --- | --- | --- | --- | --- | --- | --- | --- | --- | --- | --- | --- |
|  |  | **Search** | **NB-N** | **NB-K** | **C4.5Tree** | **1-NN** | **3-NN** | **5-NN** | **7-NN** | **Mean** | |  |
| **CVER** | **tooth-diet** | RS | 0.19 | 0.24 | 0.29 | 0.17 | 0.24 | 0.19 | 0.22 | 0.22 | |  |
|  |  | BFS | 0.14 | 0.14 | 0.19 | 0.09 | 0.14 | 0.14 | 0.12 | 0.14 | |  |
|  |  | RBFS | 0.12* | 0.14 | 0.09 | **0.07** | 0.10 | **0.07*** | 0.14 | 0.10 | |  |
|  | **toothrow-diet** | RS | 0.37 | 0.34 | 0.31 | 0.22 | 0.31 | 0.37 | 0.34 | 0.32 | |  |
|  |  | BFS | 0.26 | 0.15* | 0.29 | 0.20 | 0.30 | 0.31 | 0.26 | 0.25 | |  |
|  |  | RBFS | 0.17 | 0.16 | 0.20 | **0.09*** | 0.11 | 0.23 | 0.27 | 0.18 | |  |
|  | **mixed-diet** | RS | 0.39 | 0.42 | 0.40 | 0.34 | 0.32 | 0.35 | 0.36 | 0.37 | |  |
|  |  | BFS | 0.27* | 0.27 | 0.22 | **0.15*** | 0.30 | 0.20 | 0.24 | 0.24 | |  |
|  | **tooth-morph** | RS | 0.17 | 0.17 | 0.26 | 0.12 | 0.19 | 0.17 | 0.16 | 0.18 | |  |
|  |  | BFS | 0.15 | 0.14 | 0.12 | **0.06*** | 0.17 | 0.10 | 0.12 | 0.12 | |  |
|  |  | RBFS | 0.13 | 0.11* | 0.12 | 0.07 | 0.09 | 0.10 | 0.12 | 0.11 | |  |
|  | **toothrow-morph** | RS | 0.18 | 0.19 | 0.27 | 0.11 | 0.09 | 0.11 | 0.14 | 0.16 | |  |
|  |  | BFS | 0.08 | 0.11 | 0.17 | 0.06 | 0.11 | 0.09 | 0.10 | 0.10 | |  |
|  |  | RBFS | 0.11 | 0.07* | **0.03*** | **0.03** | **0.03** | 0.08 | 0.14 | 0.07 | |  |
| **Size** | **tooth-diet** | RS | 3 | 15 | 1 | 46 | 6 | 16 | 1 | 13 | |  |
|  |  | BFS | 6 | 9 | 4 | 5 | 4 | 3 | 5 | 5 | |  |
|  |  | RBFS | 9* | 11 | 5 | **18** | 17 | **13*** | 19 | 13 | |  |
|  | **toothrow-diet** | RS | 28 | 14 | 14 | 8 | 5 | 8 | 76 | 22 | |  |
|  |  | BFS | 9 | 8* | 2 | 6 | 2 | 2 | 4 | 5 | |  |
|  |  | RBFS | 5 | 7 | 5 | **9*** | 8 | 26 | 27 | 12 | |  |
|  | **mixed-diet** | RS | 3 | 3 | 100 | 15 | 25 | 30 | 30 | 29 | |  |
|  |  | BFS | 8* | 8 | 8 | **12*** | 3 | 7 | 9 | 8 | |  |
|  | **tooth-morph** | RS | 12 | 12 | 46 | 10 | 17 | 30 | 10 | 20 | |  |
|  |  | BFS | 5 | 6 | 3 | **6*** | 7 | 8 | 8 | 6 | |  |
|  |  | RBFS | 9 | 10* | 3 | 15 | 15 | 10 | 8 | 10 | |  |
|  | **toothrow-morph** | RS | 5 | 6 | 8 | 9 | 13 | 11 | 19 | 10 | |  |
|  |  | BFS | 8 | 4 | 2 | 6 | 9 | 4 | 7 | 6 | |  |
|  |  | RBFS | 6 | 13* | **9*** | **10** | **13** | 21 | 9 | 12 | |  |
| **Time** | **tooth-diet** | RS | 00:09 | 00:38 | 00:24 | 00:06 | 00:06 | 00:07 | 00:07 | 14 | |  |
|  |  | BFS | 00:16 | 01:15 | 00:28 | 00:12 | 00:09 | 00:08 | 00:12 | 23 | |  |
|  |  | RBFS | 03:22:42* | 10:48:05 | 12:50:43 | **02:08:27** | 01:58:22 | **01:38:54*** | 01:53:21 | 04:57:14 | |  |
|  | **toothrow-diet** | RS | 00:09 | 00:25 | 00:20 | 00:04 | 00:04 | 00:05 | 00:05 | 10 | |  |
|  |  | BFS | 00:30 | 00:53* | 00:14 | 00:09 | 00:06 | 00:07 | 00:08 | 18 | |  |
|  |  | RBFS | 02:39:31 | 06:16:56 | 09:09:53 | **01:24:34*** | 01:26:27 | 01:21:40 | 01:23:31 | 02:28:27 | |  |
|  | **mixed-diet** | RS | 00:16 | 01:14 | 01:09 | 00:21 | 00:20 | 00:19 | 00:21 | 34 | |  |
|  |  | BFS | 00:33* | 02:04 | 02:22 | **01:37*** | 00:17 | 00:41 | 00:51 | 01:12 | |  |
|  | **tooth-morph** | RS | 00:13 | 00:34 | 00:43 | 00:08 | 00:09 | 00:09 | 00:09 | 18 | |  |
|  |  | BFS | 00:17 | 00:49 | 00:32 | **00:25*** | 00:21 | 00:37 | 00:24 | 29 | |  |
|  |  | RBFS | 03:00:42 | 08:24:59* | 08:26:30 | 01:31:44 | 01:41:54 | 01:42:25 | 01:54:09 | 03:48:55 | |  |
|  | **toothrow-morph** | RS | 00:06 | 00:21 | 00:17 | 00:03 | 00:04 | 00:04 | 00:06 | 9 | |  |
|  |  | BFS | 00:27 | 00:19 | 00:16 | 00:12 | 00:17 | 00:09 | 00:15 | 16 | |  |
|  |  | RBFS | 03:31:29 | 13:37:19* | **15:16:57*** | **03:04:21** | **03:05:42** | 03:00:24 | 02:58:23 | 06:22:05 | |  |
