# Supplementary material for: Automated 3D Phenotype Analysis Using Data Mining
Source: PLoS One. 2008 Mar 5;3(3):e1742. doi: 10.1371/journal.pone.0001742 (PMC2254194; doi:10.1371/journal.pone.0001742)
Supplement: Table S3 — Feature names and IDs. (0.09 MB DOC) [file pone.0001742.s003.doc]

**Supplementary Information – Table S3**

Supplementary information for Plyusnin et al. (2008), Automated 3D phenotype analysis using data mining.

**Table S3.** Feature names and IDs.

| **ID** | **Name** |
| --- | --- |
| 1 | sectionAreas -10 -5 -1 |
| 2 | sectionAreas -10 -5 -2 |
| 3 | sectionAreas -10 -5 -3 |
| 4 | sectionAreas -10 -5 -4 |
| 5 | sectionAreas -10 -5 -5 |
| 6 | sectionAreas -10 -5 -6 |
| 7 | sectionAreas -10 -5 -7 |
| 8 | sectionAreas -10 -5 -8 |
| 9 | sectionAreas -10 -5 -9 |
| 10 | sectionAreas -10 -5 -10 |
| 11 | sectionConv -10 -5 -1 |
| 12 | sectionConv -10 -5 -2 |
| 13 | sectionConv -10 -5 -3 |
| 14 | sectionConv -10 -5 -4 |
| 15 | sectionConv -10 -5 -5 |
| 16 | sectionConv -10 -5 -6 |
| 17 | sectionConv -10 -5 -7 |
| 18 | sectionConv -10 -5 -8 |
| 19 | sectionConv -10 -5 -9 |
| 20 | sectionConv -10 -5 -10 |
| 21 | MROPC -4 -0.001 -9 |
| 22 | MROPC -4 -0.002 -9 |
| 23 | MROPC -4 -0.004 -9 |
| 24 | MROPC -4 -0.006 -9 |
| 25 | MROPC -4 -0.008 -9 |
| 26 | MROPC -4 -0.01 -9 |
| 27 | MROPC -4 -0.02 -9 |
| 28 | MROPC -4 -0.04 -9 |
| 29 | MROPC -4 -0.06 -9 |
| 30 | MROPC -4 -0.08 -9 |
| 31 | MROPC -4 -0.1 -9 |
| 32 | MROPC -5 -0.001 -9 |
| 33 | MROPC -5 -0.002 -9 |
| 34 | MROPC -5 -0.004 -9 |
| 35 | MROPC -5 -0.006 -9 |
| 36 | MROPC -5 -0.008 -9 |
| 37 | MROPC -5 -0.01 -9 |
| 38 | MROPC -5 -0.02 -9 |
| 39 | MROPC -5 -0.04 -9 |
| 40 | MROPC -5 -0.06 -9 |
| 41 | MROPC -5 -0.08 -9 |
| 42 | MROPC -5 -0.1 -9 |
| 43 | MROPC -6 -0.001 -9 |
| 44 | MROPC -6 -0.002 -9 |
| 45 | MROPC -6 -0.004 -9 |
| 46 | MROPC -6 -0.006 -9 |
| 47 | MROPC -6 -0.008 -9 |
| 48 | MROPC -6 -0.01 -9 |
| 49 | MROPC -6 -0.02 -9 |
| 50 | MROPC -6 -0.04 -9 |
| 51 | MROPC -6 -0.06 -9 |
| 52 | MROPC -6 -0.08 -9 |
| 53 | MROPC -6 -0.1 -9 |
| 54 | MROPC -7 -0.001 -9 |
| 55 | MROPC -7 -0.002 -9 |
| 56 | MROPC -7 -0.004 -9 |
| 57 | MROPC -7 -0.006 -9 |
| 58 | MROPC -7 -0.008 -9 |
| 59 | MROPC -7 -0.01 -9 |
| 60 | MROPC -7 -0.02 -9 |
| 61 | MROPC -7 -0.04 -9 |
| 62 | MROPC -7 -0.06 -9 |
| 63 | MROPC -7 -0.08 -9 |
| 64 | MROPC -7 -0.1 -9 |
| 65 | MROPC -8 -0.001 -9 |
| 66 | MROPC -8 -0.002 -9 |
| 67 | MROPC -8 -0.004 -9 |
| 68 | MROPC -8 -0.006 -9 |
| 69 | MROPC -8 -0.008 -9 |
| 70 | MROPC -8 -0.01 -9 |
| 71 | MROPC -8 -0.02 -9 |
| 72 | MROPC -8 -0.04 -9 |
| 73 | MROPC -8 -0.06 -9 |
| 74 | MROPC -8 -0.08 -9 |
| 75 | MROPC -8 -0.1 -9 |
| 76 | MROPC -9 -0.001 -9 |
| 77 | MROPC -9 -0.002 -9 |
| 78 | MROPC -9 -0.004 -9 |
| 79 | MROPC -9 -0.006 -9 |
| 80 | MROPC -9 -0.008 -9 |
| 81 | MROPC -9 -0.01 -9 |
| 82 | MROPC -9 -0.02 -9 |
| 83 | MROPC -9 -0.04 -9 |
| 84 | MROPC -9 -0.06 -9 |
| 85 | MROPC -9 -0.08 -9 |
| 86 | MROPC -9 -0.1 -9 |
| 87 | MROPC -10 -0.001 -9 |
| 88 | MROPC -10 -0.002 -9 |
| 89 | MROPC -10 -0.004 -9 |
| 90 | MROPC -10 -0.006 -9 |
| 91 | MROPC -10 -0.008 -9 |
| 92 | MROPC -10 -0.01 -9 |
| 93 | MROPC -10 -0.02 -9 |
| 94 | MROPC -10 -0.04 -9 |
| 95 | MROPC -10 -0.06 -9 |
| 96 | MROPC -10 -0.08 -9 |
| 97 | MROPC -10 -0.1 -9 |
| 98 | relief |
| 99 | D2dist -100000 -mean |
| 100 | D2dist -100000 -std |
